# Liquid Biopsy Based Bladder Cancer Diagnostic by Machine Learning

**DOI:** 10.3390/diagnostics15040492

**Published:** 2025-02-18

**Authors:** Ērika Bitiņa-Barlote, Dmitrijs Bļizņuks, Sanda Siliņa, Mihails Šatcs, Egils Vjaters, Vilnis Lietuvietis, Miki Nakazawa-Miklaševiča, Juris Plonis, Edvīns Miklaševičs, Zanda Daneberga, Jānis Gardovskis

**Affiliations:** 1 Institute of Oncology and Molecular Genetics, Riga Stradins University, LV-1002 Riga, Latvia; 2 Department of Urology, Paul Stradins Clinical University Hospital, LV-1002 Riga, Latvia; 3 Institute of Applied Computer Systems, Faculty of Computer Science, Information Technology and Energy, Riga Technical University, LV-1048 Riga, Latvia; 4 Clinic of Urology and Oncological Urology, Riga East University Hospital, LV-1079 Riga, Latvia; 5 Department of Biology and Microbiology, Riga Stradins University, LV-1007 Riga, Latvia; 6 Department of Surgery, Riga Stradins University, LV-1002 Riga, Latvia; 7 Department of Surgery, Paul Stradins Clinical University Hospital, LV-1002 Riga, Latvia

**Keywords:** artificial intelligence, machine learning, miRNAs, bladder cancer, multi-modal data, biomarker, liquid biopsy, biofluids, urine exosomes

## Abstract

**Background/Objectives**: The timely diagnostics of bladder cancer is still a challenge in clinical settings. The reliability of conventional testing methods does not reach desirable accuracy and sensitivity, and it has an invasive nature. The present study examines the application of machine learning to improve bladder cancer diagnostics by integrating miRNA expression levels, demographic routine laboratory test results, and clinical data. We proposed that merging these datasets would enhance diagnostic accuracy. **Methods**: This study combined molecular biology methods for liquid biopsy, routine clinical data, and application of machine learning approach for the acquired data analysis. We evaluated urinary exosome miRNA expression data in combination with patient test results, as well as clinical and demographic data using three machine learning models: Random Forest, SVM, and XGBoost classifiers. **Results**: Based solely on miRNA data, the SVM model achieved an ROC curve area of 0.75. Patient analysis’ clinical and demographic data obtained ROC curve area of 0.80. Combining both data types enhanced performance, resulting in an F1 score of 0.79 and an ROC of 0.85. The feature importance analysis identified key predictors, including erythrocytes in urine, age, and several miRNAs. **Conclusions**: Our findings indicate the potential of a multi-modal approach to improve the accuracy of bladder cancer diagnosis in a non-invasive manner.

## 1. Introduction

The current one-size-fits-all approach in cancer medicine is rapidly moving towards precision oncology, tailoring diagnostics and subsequent therapies to the unique characteristics of each individual patient. The ninth most common malignancy in the world is bladder cancer [1]. It is observed that bladder cancer occurs three times more often in men than in women. Approximately 70% of patients with bladder cancer are over 65 years of age [2]. Even though the number of incidents of disease in Europe is declining, there is a slight increase in the Baltic countries and in the countries of Southern and Eastern Europe. European males have the highest recorded mortality rates worldwide, especially in Eastern Europe, Southern Europe, and the Baltic States [1,3].

Early and accurate diagnosis of bladder cancer is crucial to estimate prognosis, predict response to treatment, and monitor recurrence. At present, the standard clinical diagnostics for bladder cancer detection include invasive cystoscopy combined with non-invasive cytology [4]. Approximately in 30% of the cases, the diagnosis is delayed and has a poor prognosis because muscle-invasive BC rapidly progresses and metastasises; therefore, it correlates with a high mortality rate despite the new therapeutic strategies [5].

Urinary cytology has high sensitivity in high-grade tumours, including carcinoma in situ, but may be negative in low-grade tumours. Although the development of new technologies has provided fluorescent and narrow-band imaging, there are several issues that are associated with the fact that cystoscopy is a relatively expensive and invasive diagnostic method. Furthermore, it is not usually used in daily practice in patients without lower urinary tract symptoms or haematuria [6]. European guidelines consider the use of some specific biomarkers for better diagnosis and disease recurrence control. These are the UroVysion (FISH), microsatellite analysis, Immunocyt/uCyt +, Nuclear matrix Protein 22, BTA stat, BTA TRAK, and Cytokeratins [4]. The main problem of using noninvasive urine tests in clinical practice is that their diagnostic specificity is inferior to urinary cytology tests [7]. In a prospective multicentre study, previously described urinary biomarkers of bladder cancer such as BTA stat, NMP22 BladderChek, UBC Rapid Test, and CancerCheck UBC rapid VISUAL compared to cytology for all stages of bladder cancer achieved a sensitivity of 76.7%, 33.0%, 72.2%, 47.2%, and 55.8% and a specificity of 67.9%, 95.5%, 79.4%, 94.4%, and 83.7%. However, the area under the curve (AUC) was less than 0.7 for almost all tests, except for BTA stat, which had an AUC of 0.722, which also sounds like a poor result [8]. This leads to attempts to identify new biomarkers for bladder cancer diagnostics that could be used as noninvasive diagnostic tools and could be helpful for early detection of disease and its recurrence with high specificity and sensitivity.

Such biomarkers could be miRNAs that bind to the 3′-UTR of target mRNAs and thus post-transcriptionally regulate gene expression, which leads to the degradation of the target mRNA or inhibition of their translation [9]. They participate in the regulation of various biological functions in norm and cancer development and progression to metastasis [10]. These kinds of markers could be useful as miRNAs are stable within urine and require little handling care. Moreover, due to their small size, they are more stable against nuclease degradation [11,12].

A number of studies have been performed with the aim to identify miRNA-based biomarkers for bladder cancer diagnostics. Unfortunately, these studies did not lead to overlapping results: almost all miRNAs were found only in one or two studies (with the only exception being miR-96-5p) [13,14]. Despite this variability, a promising new study developed a screening model using serum miRNAs and machine learning algorithms, demonstrating high accuracy in early bladder cancer detection across a large mixed-cohort study [15]. Similar findings have been reported in other studies, suggesting the potential of miRNA as biomarkers [16].

Further research has identified miRNA editing events in recurrent bladder cancer, implying their involvement in disease progression and potential as therapeutic targets [17]. Additionally, miRNA expression signatures have been explored for their ability to estimate survival time in bladder cancer patients. A method called BLC-SVR, based on support vector regression and an optimal feature selection algorithm, was developed to identify a robust set of miRNAs associated with survival in bladder cancer. Urinary miRNAs have also been analysed as diagnostic tools for bladder cancer, showing promise in distinguishing between cancerous and non-cancerous conditions [13].

While these results are encouraging, blood sampling remains invasive and uncomfortable for patients, highlighting the advantages of urinalysis as a non-invasive, easy-to-obtain method that requires no special medical personnel. In our previous study, we identified a number of miRNAs in urinal exosomes that are presented at higher levels in bladder cancer patients compared to age and sex matched controls [18].

Ongoing research aims to address the shortcomings of previous studies by incorporating machine learning and artificial intelligence. One effective approach is the evolutionary learning method, Cancer*Sig*, proposed by Sathipati et al. [19], which distinguishes between early-stage and advanced-stage cancers using miRNA expression profiles. By combining the IBCGA feature selection algorithm, SVM classifier, and pan-analysis of miRNA signatures, this method achieves impressive accuracy metrics across 15 different cancer types [19].

There is still much to explore in order to find better and less invasive diagnosis methods for bladder cancer that can be integrated into clinical practice. One potential approach involves combining various data formats, as demonstrated by Moisoiu et al. [20] in their study on liquid urine biopsies. Their research, which utilised machine learning alongside a combination of miRNA profiling and SERS profiling, indicated improved results for point-of-care diagnosis and molecular stratification of bladder cancer [20].

This study examines the application of machine learning to improve bladder cancer diagnostics by integrating miRNA expression levels and demographic, routine laboratory test results and clinical data. We proposed that merging these datasets in an AI-trained algorithm would enhance the diagnostic accuracy of bladder cancer diagnosis in a non-invasive manner.

## 2. Materials and Methods

### 2.1. Study Group and Inclusion Criteria

From 2019 to 2024, prospective enrolment occurred at Pauls Stradins Clinical University Hospital and Riga East University Hospital for individuals suspected of having bladder cancer (BCa) and control subjects. All patients and control group participants have consented to take part in this study.

The criteria for the bladder cancer group required participants to be over the age of 50 years with pathohistological proven primary or recurrent bladder cancer after performing transurethral resection of bladder (TURB) or cystectomy, without any other existing oncological conditions or prior upper urinary tract cancers, and have available medical records. Participants were categorised and staged based on the TNM classification and WHO criteria. Any patient without a confirmed pathohistological diagnosis of bladder cancer had their urine sample excluded from the analysis.

The control group included patients who met the following criteria: they should not have any history of malignancy, urine analyses should be without signs of inflammation or infection, and they should not have significant abnormalities in their blood tests.

For both groups, exclusion criteria were autoimmune disease, diabetes mellitus, or any infections other than urinary tract infections related to bladder cancer.

**Figure 1:**
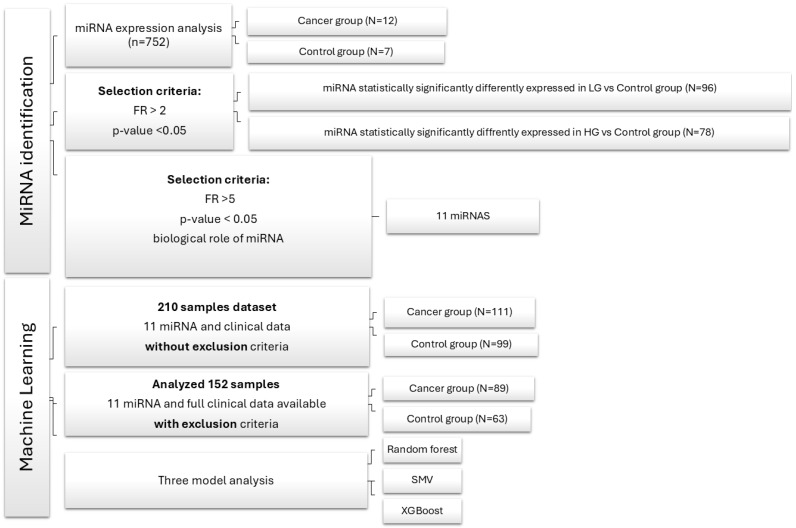
Flowchart of data analysis. FR—fold regulation; SMV—support vector machine; XGBoost—extreme gradient boosting.

Initially, our database consisted of 210 patients, 111 in the cancer group and 99 in the control group, who met the exclusion criteria. For further data analysis, patients without exclusion criteria were selected, resulting in a total of 89 patients in the cancer group and 63 in the control group. See Figure 1 for a detailed description.

### 2.2. Processing of Urine Samples, Exosome Isolation, and RT-qPCR

Spontaneous voided urine samples were collected for the bladder cancer and control groups before the TURB procedure or any surgical intervention. Urine sample processing was performed within 24 h of urine collection by centrifuging the samples at room temperature at 3.0 rcf (4.4 rpm) for 5 min and 30 s. The resulting supernatant was then stored at −80 °C until it was needed for analysis.

Prior to isolating miRNA, exosome isolation was performed according to the protocol outlined by the miRCRURY Exosome Cell/Urine/CSF Kit (Qiagen, Hilden, Germany, Cat. No./ID: 76743). The urine samples were thawed on ice and subjected to a second centrifugation for 10 min at 10,000× *g* at room temperature to eliminate any remaining cellular debris. Next, exosomes were enriched and isolated from 1960 µL of the urine supernatant. The miRNeasy Mini Kit (Qiagen, Germany, Cat. No./ID: 17004) was then used to extract miRNA and total RNA from the urine exosomes, following the manufacturer’s instructions. Each sample was spiked with a synthetic UniSp2, UniSp4, and UniSp5 RNA mix (Qiagen, Germantown, MD, USA, Cat. No./ID: 339390) for both isolation and RT-PCR quality control, followed by RNA purification and elution in 50 µL RNase-free water. For reverse transcription and polyadenylation of miRNA to cDNA, the MiRCURY LNA RT Kit (Qiagen, USA, Cat. No./ID: 339340) was employed, incorporating a UniSp6 spike-in. The incubation was carried out using the peqSTAR thermal cycler at 42 °C for 60 min, followed by 95 °C for 5 min, and subsequently held at 4 °C.

The quantification of the preselected miRNAs was conducted using the pre-designed miRCURY LNA miRNA Custom PCR Panel YCA34464 (Qiagen, USA, Cat. No./ID: 339322) and the miRCURY LNA SYBR Green PCR Kit (Qiagen, USA, Cat. No./ID: 339347). The list of selected miRNAs and the configuration of the plate are provided in Table 1. The qPCR for the miRNA panel was performed with the Applied Biosystems^®^ ViiA™ 7 Real-Time PCR system, following the manufacturer’s protocol. An automatic threshold and baseline were applied for all miRNAs to record the CT values.

### 2.3. RT-qPCR Data Analysis

The analysis of miRNA panels was conducted using miRCURY miRNA PCR Data Analysis v1.0. Data normalisation was achieved through a global mean normalisation approach. Differences in miRNA expression levels between the control and cancer groups were assessed by calculating *p*-values based on a Student’s *t*-test applied to the replicate 2^−ΔCT^ values for each miRNA in both groups. The method for calculating the *p*-values involved a parametric, two-sample equal variance, unpaired, two-tailed distribution. The GeNorm module, included as part of miRCURY miRNA PCR Data Analysis v1.0, was employed to identify internal control miRNAs that displayed expression levels closely correlating with the global mean CT values and that best matched the average CT value.

### 2.4. Machine Learning

All three classifiers (Random Forest, SVM, XGBoost) were applied to both miRNA and laboratory data, as well for the combined set, by evaluating feature importance and model performance. Parameter finetuning allowed for a maximising of the accuracy. The leave-one-out method allowed the use of an almost full dataset for training and keeping the model robust. All contributions made by the AI tool were thoroughly reviewed and approved by medical experts to ensure accuracy and adherence to scientific standards.

Random Forest combines many decision trees to reduce overfitting and capture varied patterns in the data. Support Vector Machine focuses on finding the best boundary between classes and can model nonlinear relationships effectively. XGBoost refines its decision trees step by step, achieving strong accuracy and handling missing values well. Each of these algorithms can adapt to a moderate sample size and is widely used in biomedical studies because it balances flexibility, robustness, and performance when working with complex biological features.

This approach aligns with recent studies that highlight the potential of miRNAs as biomarkers for bladder cancer diagnosis and prognosis [13,15,17].

## 3. Results

### 3.1. Study Group

In the beginning, we had a dataset of 210 patients with exclusion criteria as follows: autoimmune disease, diabetes mellitus, any infections other than urinary tract infections related to bladder cancer, any history of malignancy, urine analyses with signs of inflammation or infection, and no significant abnormalities in their blood tests. For further data analysis to validate the possibility of employing selected miRNAs (see below) as biomarkers for non-invasive bladder cancer, diagnostic urine samples were collected from 152 samples without exclusion criteria and complete clinical data information; 89 bladder cancer patients and 63 healthy controls, see Figure 1. For more detailed information on the cohorts, see Table 2.

### 3.2. Selection of Putative Biomarkers

As described earlier (B-B, 2024), differentially presented miRNAs with a cut-off of over 2-fold regulation (FR) or under -2 FR in urinal exosomes from low-grade and high-grade bladder cancer patients as compared to control subjects were analysed. The number of miRNAs corresponding to these cut-off values was 96 miRNAs in the low-grade group (93 up-regulated and 3 down-regulated) and 78 in the high-grade group (72 up-regulated and 6 down-regulated). Among these differentially presented miRNAs in the exosome, 42 miRNAs were found in both the LG and HG groups, and these miRNAs were all up-regulated in cancer patients [18].

From this pool of miRNAS, candidate molecules were selected on following principles: (i) at least 5-fold increase in urinal exosomes either from low- or high-grade bladder cancer patients as compared to control subjects; (ii) small *p*-value and (iii) target genes. One exception was hsa-mir-429, which was increased in high-grade samples only 3.19-fold due to a very low *p*-value (0.00006); see Table 3. UniSp2, UniSp3, and UniSp5 were included as spikes. Hsa-miR-1260a were used for normalisation.

### 3.3. Machine Learning Model Development

In this study, we studied three classifiers to diagnose bladder cancer using a combination of miRNA expression levels and clinical patient data. We included a total of 152 patients, comprising 89 bladder cancer patients and 63 control subjects. The features used in the model encompassed both miRNA expression levels and clinical analyses; in total, there were 24 features.

**Figure 2:**
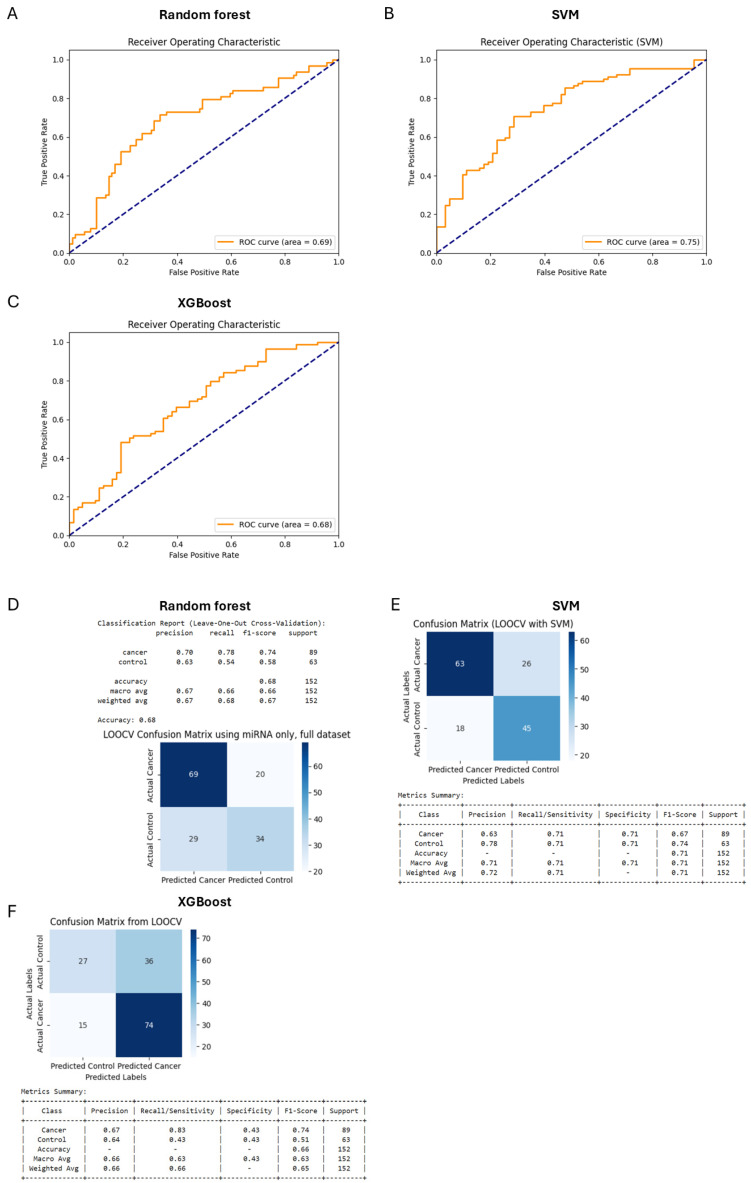
Areas under curve (AUC) after analysing miRNA levels by Random Forest (**A**), SMV (**B**), and XGBoost model (**C**). Confusion matrix for the Random Forest (**D**), SMV (**E**), and XGBoost (**F**) model trained with differentially expressed miRNAs.

**Figure 3:**
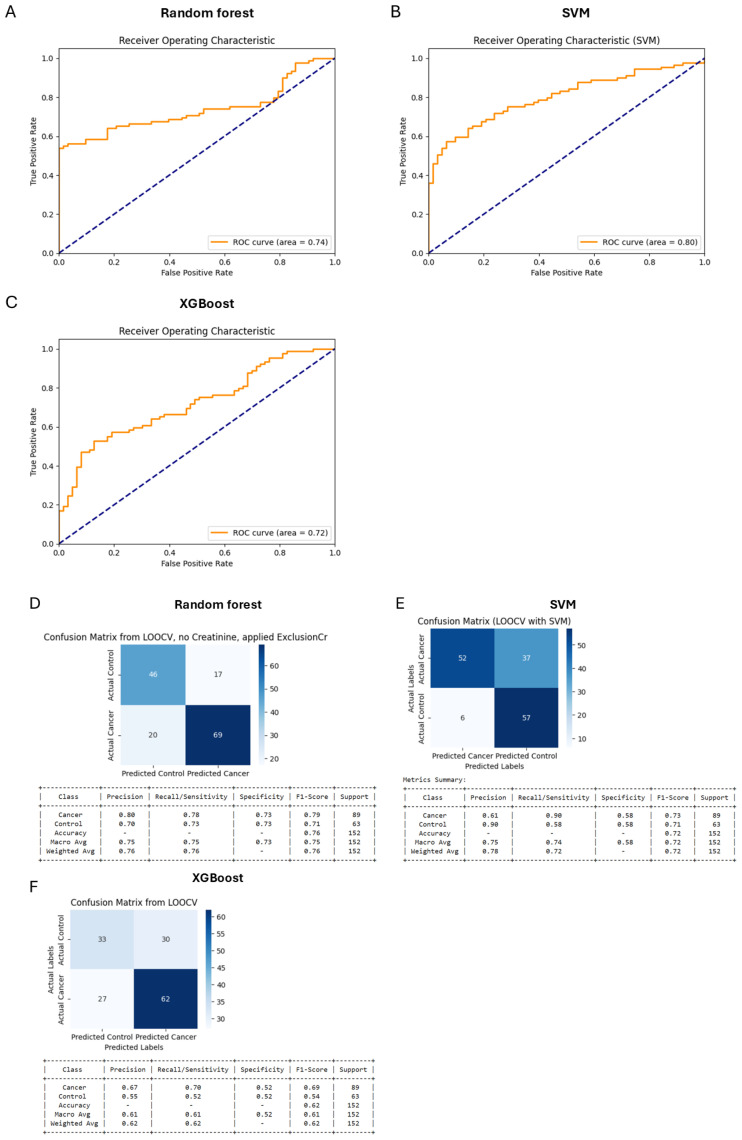
Areas under curve (AUC) after analysing the clinical data set by Random Forest (**A**), SMV (**B**) and XGBoost model (**C**). Confusion matrix for the Random Forest (**D**), SMV (**E**), and XGBoost (**F**) model trained with a clinical data set.

**Figure 4:**
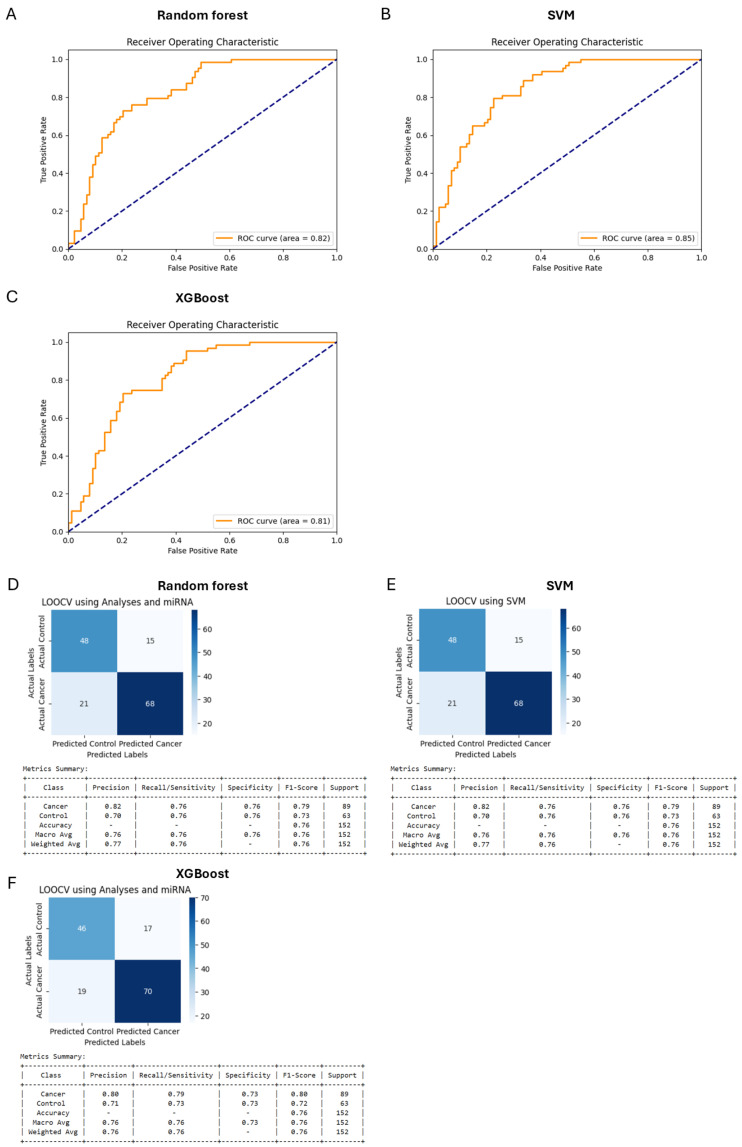
Areas under curve (AUC) after analysing miRNA levels and the clinical data set combined by Random Forest (**A**), SMV (**B**), and XGBoost model (**C**). Confusion matrix for the Random Forest (**D**), SMV (**E**), and XGBoost (**F**) model trained with miRNA levels and the clinical data set combined.

**Figure 5:**
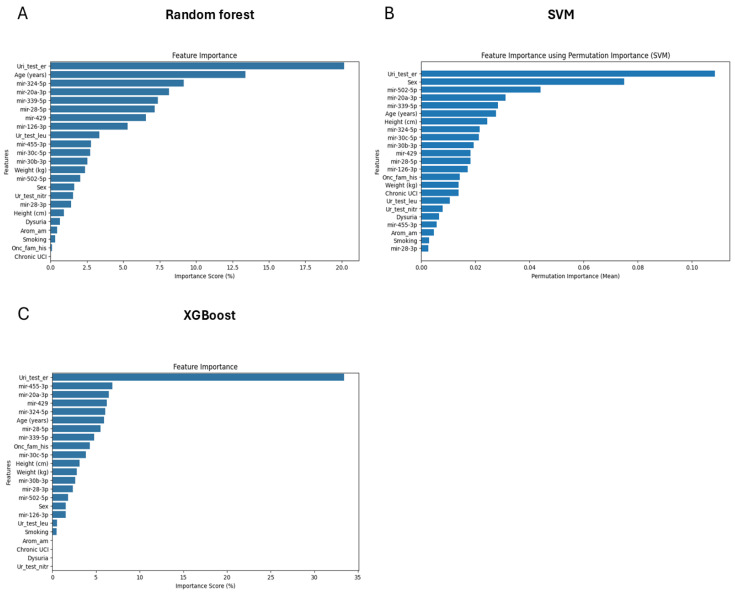
Feature importance analysis in a combined data set by three machine learning models: Random Forest (**A**), SVM (**B**), and XGBoost (**C**). Uri_test_er—eritocituria in dipstick urine analysis; Ur_test_leu—leucocituria in dipstick urine analysis; Ur-test_nitr—positive nitrite test in dipstick urine analysis; Arom_am—exposure to aromatic amines, Onc-farm_hist—oncological history in family; Chronic UCI—chronic urinary tract infection.

Initially, separate models using only miRNA data (Figure 2) and only clinical analyses data (Figure 3) were developed to evaluate their individual predictive capabilities. The combined data model (Figure 4), however, outperformed both separate models, justifying the integration approach. This combined dataset captured the multifactorial nature of bladder cancer, thereby improving diagnostic accuracy. Analysis of feature importance showed that the most discriminating factors are the number of urinal erythrocytes and age among clinical factors and miRNAs such as hsa-miRNA-28-5p, hsa-miRNA-324-5p, hsa-miRNA-429, and hsa-miRNA-20a-3p and as key discriminators between cancer patients and non-cancer control subjects (Figure 5).

To maximise the models’ performance, we performed hyperparameter tuning, focusing on the following parameters: n_estimators and max_depth for Random Forest and XGBoost; C and gamma for SVM. For n_estimators, ranges from 5 to 105 were tested in increments of 20 during the initial coarse search and then refined to 80–90 in increments of 1 for fine-tuning. For max_depth, ranges from 3 to 50 were tested in increments of 10 during the initial search and then refined to 3–10 in increments of 1. In SVM, the ranges were as follows: C [0.1..10], gamma [0.001..1].

Leave-one-out cross-validation (LOOCV) was employed to assess model performance for each parameter combination, which is advantageous for small datasets as it maximises the use of available data for training while testing on a single observation. Parallel processing was implemented to expedite the evaluation process across multiple parameter combinations, significantly reducing computational time.

Performance metrics included accuracy—the proportion of true results among the total number of cases examined—sensitivity (recall), which is the ability of the model to correctly identify patients with bladder cancer, and specificity, the ability to correctly identify control subjects without bladder cancer. The initial grid search was carried out in two stages: first, with a coarse sampling of parameter combinations and, subsequently, with a more focused fine-tuning. In the coarse search, multiple values of parameters were systematically explored in a grid, and each parameter setting was evaluated using the leave-one-out technique. This process enabled the identification of broad parameter regions where model performance, as measured by cross-validation accuracy and F1 scores, was markedly higher. Building on these insights, a finer grid was subsequently defined around the most promising intervals for both parameters. In this targeted range, step sizes were reduced to one, allowing for closer examination of parameter interactions and performance trade-offs. The outcome of this refined grid search was the selection of best-performing hyperparameters, yielding the best cross-validation results while maintaining model generalizability.

These settings provided the best balance between model complexity and performance, effectively preventing overfitting while maintaining high predictive accuracy. The shallow tree depth ensured that the trees were not overly complex, which could lead to overfitting, while a sufficient number of trees contributed to the model’s robustness.

To ensure consistency and reliability of the model’s performance, the training and evaluation process was repeated multiple times with different random states. Specifically, the model was trained and evaluated 20 times, each with a different random seed. Performance metrics were averaged across all repetitions, confirming that the model’s performance was not dependent on a particular random seed used in the algorithm.

## 4. Discussion

On one hand, the miRNA pool seems to be a very good source of biomarkers for diagnostic purposes. These molecules are small, stable, easy to work with, and are presented in all tissues and biological liquids and reflect changes between different physiological states. On the other hand, there are huge inconsistencies in miRNA levels, and it is difficult to obtain reproducible results in different studies. In order to identify diagnostic miRNAs bladder cancer, these molecules were isolated from whole urine, supernatant, sediment, and exosomes in a number of studies [13]. As a result, more than 100 putative miRNA biomarkers were identified, but only one miRNA (miR-96/miR-96-5p) was found in three independent studies. It should be noted that among many factors influencing miRNA expression, ethnic origin may play a role [14]. These considerations lead to the necessity of more studies on the feasibility of using miRNAs as effective and reliable markers for early, non-invasive cancer diagnostic in general and bladder cancer in particular.

In the present study, we explored the diagnostic potential of seven miRNAs (hsa-miR-28-3p, hsa-miR-30b-3p, hsa-miR-339-5p, hsa-miR-502-5p, hsa-miR-20a-3p, and hsa-miR-429), which were identified as candidates in our previous study [18]. All selected miRNAs are involved in the regulation of tumour development but are not necessarily described as bladder cancer-related as yet. Thus, two different controversial roles were shown for hsa-miR-20a-3p: in melanoma cells, it was acting as an onco-suppressor [21], but in pancreatic ductal adenocarcinoma [22], tongue squamous cell carcinoma [23], and colorectal cancer [24], it was the driver of proliferation and metastasis. It was shown in a relatively small sample number that the hsa-miR-28-3p level was increased in plasma samples from patients with non-muscle invasive bladder cancer as compared to non-malignant urological diseases as a control group [25]. Contradictory results were obtained about the role of hsa-miR-502-5p; in some studies, it was promoting proliferation and invasion and inhibiting apoptosis [26,27], while in other ones, it mediated inhibition of cell proliferation and migration [28]. Increased levels of hsa-miR324-5p in urine samples were associated with poor survival of bladder cancer patients due to accelerated cancer cell proliferation and increased ability of invasion and colony formation [29,30]. Hsa-miR-429 also promotes bladder cancer cell proliferation by inhibiting CDKN2B [31]. It is important to notice that the diagnostic power of has-miR-324-5p alone to discriminate the bladder cancer group from control subjects was impressive: sensitivity = 0.878, specificity = 0.867, AUC = 0.883 [32]. Likewise, when hsa-miR-429 was used in a panel with a number of other miRNAs, high diagnostic power was achieved (sensitivity = 0.870, specificity = 1, AUC = 0.982) [33].

At present, magnetic resonance imaging (MRI) with state-of-the-art technology is being utilised more frequently for the diagnostic evaluation of bladder cancer. This imaging technique provides high-resolution anatomical details, facilitating an accurate assessment of bladder lesions and adjacent tissues [33,34]. Several studies have explored the application of machine learning techniques to radiological examinations, highlighting their potential for future advancements [35,36,37,38]. However, several notable limitations exist. The availability of the aforementioned MRI technology is often constrained due to lengthy waiting lists and high costs, which may result in delays in patient care and disease progression. It is a time-consuming procedure, with possible motion artifacts and may be uncomfortable for some patients, especially those with claustrophobia. Additionally, the use of contrast agents in MRI can pose risks, potentially increasing the likelihood of developing other medical conditions [39].

Based on meta-analysis, it was estimated that the diagnostic efficacy of urinal exosomal miRNAs was rather high: sensitivity—0.69; specificity—0.81; AUC—0.83 [40]. These values considerably exceed the diagnostic power of selected miRNA panel presented in this study—AUC = 0.75 (Figure 2B,E). Furthermore, the diagnostic power of a combination of a number of clinical parameters was better (AUC = 0.80) (Figure 3B,E). A combination of miRNA levels and clinical data led to the best result: sensitivity—0.76; specificity—0.76; AUC—0.85 (Figure 4B,E). In this study, we developed a Random Forest model that integrates miRNA expression levels with clinical patient data to enhance the diagnostic accuracy for bladder cancer. While the model demonstrated robust performance, several aspects merit further discussion. Moreover, when looking at the feature of importance (Figure 5), it is noteworthy that smoking does not rank among the top factors, despite being a widely recognised risk factor [3]. This could be explained by the fact that individual pack-years were not included here, which would better reflect the data, as people who smoked only were also included. Other features of clinical importance are consistent with those described in the literature, such as age and haematuria.

One limitation pertains to the handling of missing blood creatinine values. Filling missing data with the average value of the respective group, although a common practice, is not the most optimal solution. This method can introduce bias by underrepresenting the natural variability within the dataset and may not accurately reflect individual patient differences. Advanced data imputation methods, such as multiple imputation or model-based approaches, could potentially provide more accurate estimations of missing values by considering the relationships between variables. However, the relatively small sample size in our dataset limits the reliability of these advanced imputation techniques, as they often require larger amounts of data to model the underlying data distribution effectively [41]. Implementing sophisticated imputation methods under these conditions might lead to overfitting or introduce additional bias, thereby affecting the model’s generalizability.

Another consideration is the exploration of alternative machine learning algorithms. While the Random Forest method is well-suited for handling high-dimensional data and provides robustness against overfitting through ensemble learning [42], other AI techniques could be investigated to potentially improve model performance. Methods such as support vector machines, gradient boosting, or ensemble techniques might offer different advantages in terms of capturing complex patterns within the data. However, more complex models like deep learning architectures may not be suitable in this context due to the limited amount of data. Deep learning models typically require large datasets to train effectively and are prone to overfitting when applied to smaller datasets, which could diminish their predictive accuracy on unseen data.

Future studies should also consider the use of advanced feature selection and dimensionality reduction techniques to enhance model interpretability and performance. Additionally, investigating the biological relevance of the most influential features identified by the model could provide insights into the molecular mechanisms underlying bladder cancer and potentially uncover novel biomarkers for early detection.

Despite the promising advances in the use of machine learning and urinary miRNAs in bladder cancer diagnosis, several limitations remain due to the lack of clinical validation. The next phase of this research should include a double-blind study designed to further validate the screening model, followed by a multicentre clinical trial to evaluate its performance in real-world clinical practice. This trial should include longitudinal participant observation to monitor the evolution of the model’s accuracy and generalizability in a dynamic healthcare environment.

Given the demographic differences and comorbidities that are particularly relevant to bladder cancer patients, many of whom are elderly, there is an urgent need for studies that include individuals with specific exclusion criteria, such as chronic comorbidities such as diabetes and autoimmune diseases, which were not addressed in this initial phase. In addition, the developed model requires further validation to confirm its efficacy in different patient profiles. A notable strength of our study is the isolation of miRNAs from exosomes, addressing a major shortcoming observed in previous studies and potentially improving the robustness of the diagnostic approach. The implementation of a multicentre study design would increase the external validity of the findings and facilitate the integration of diverse patient demographics, further strengthening the clinical applicability of the model.

## 5. Conclusions

In this study, we demonstrated that clinical data synergises with urine miRNA profiling for a better BC diagnostic. Our findings indicate the potential of a multi-modal data approach to improve the accuracy of bladder cancer diagnosis in a non-invasive manner and led to the best result: sensitivity—0.76; specificity—0.76; AUC—0.85.

SVM classifier turned out to be better than Random Forest and XGBoost. This could be explained by the fact that SVM focuses on the boundary between classes and often performs well with smaller datasets, making it less prone to overfitting. By concentrating on the most relevant points in the data, it can detect small differences in the expression of the selected miRNAs, leading to improved classification results.

## Figures and Tables

**Table 1:** Configuration of the miRCURY LNA miRNA Custom PCR Panel.

Position	Assay Name
A01–A24	UniSp6
B01–B24	UniSp3
C01–C24	UniSp2
D01–D24	UniSp5
E01–E24	hsa-miR-30c-5p
F01–F24	hsa-miR-126-3p
G01–G24	hsa-miR-1260a
H01–H24	hsa-miR-20a-3p
I01–I24	hsa-miR-28-3p
J01–J24	hsa-miR-28-5p
K01–K24	hsa-miR-30b-3p
L01–L24	hsa-miR-324-5p
M01–M24	hsa-miR-339-5p
N01–N24	hsa-miR-429
O01–O24	hsa-miR-455-3p
P01–P24	hsa-miR-502-5p

**Table 2:** Clinical–pathological parameters of the study cohorts.

Variable		Study Groups	
		Control (N = 63)	Cancer (N = 89)
Age (years)			
	Median	66	74
Gender			
	Male	39	70
	Female	24	19
Pathological stage			
	≤pT1	-	80
	≥pT2	-	9
Grade			
	LG	-	52
	HG	-	37
Eritrocituria			
	Yes	4	50
	No	59	39
Smoking history	Present/Past	30	47
	Never	33	42

N, number; LG, low grade; HG, high grade.

**Table 3:** miRNAS selected for validation as putative biomarkers from the identification steps.

miRNA	Target Gene/s	Low-Grade vs. Control		High-Grade vs. Control	
		FR	*p*	FR	*p*
hsa-miR-20a-3p	*PTEN*, *EGR2*, *BID*, *SMO*, *NR4A3*	5.11	0.01117	7.36	0.005637
hsa-miR-28-5p	*TP53*, *MAPK1*, *IGF1*, *STAT5B*, *IL34*, *CDKN1A*, *MPL*, *N4BP1*, *OTUB1*, *TEX261*	5.3	0.039468	9.95	0.000754
hsa-miR-30b-3p	*BECN1*, *RORC*	8.57	0.001235	1.58	0.236102
hsa-miR-126-3p	*AKT1*, *CADM1*, *EZH2*, *ROCK1*, *HOXA9*, *SPRED1*, *PLK2*, *RGS3*, *TOM1*, *VEGFA*, *CRK*, *IRS1*, *VEGFA*, *PIK3R2*, *VCAM1*, *IRS1*, *EGFL7*, *SOX2*, *PTPN7*, *DNMT1*, *KRAS*, *SLC7A5*, *IGFBP2*, *PITPNC1*, *MERTK*, *TEK*, *PIK3CG*, *CXCL12*, *ADAM9*, *MMP7*, *SIRT1*, *CRKL*, *PGR*, *CXCR4*, *RHOU*, *LRP6*, *NFKBIA*, *FOXO3*, *BCL2*, *ADGRE5*, *TCF4*, *LRP6*, *ADM*	5.62	0.002687	21.35	0.015415
hsa-miR-324-5p	*MTFR1*, *CD274*, *ETS1*, *GLI1*, *SMO*, *SP1*	12.24	0.007172	11.47	0.009456
hsa-miR-429	*CRKL*, *FSCN1*, *IL4*, *HIF1A*, *DLC1*, *SP1*, *MALAT1*, *VEGFA*, *ZEB2*, *RERE*, *ZEB1*, *MYB*, *BCL2*, *XIAP*, *SOX2*, *WASF3*, *ZFPM2*, *OSTF1*, *KLHL20*, *PTPRD*, *ELMO2*, *ERBIN*, *BAP1*, *WDR37*, *VAC14*, *TCF7L1*, *HOXB5*, *RASSF2*, *RIN2*, *KLF11*, *SEPT7*, *SHC1*, *MYC*, *DNMT1*, *EZH2*, *TIMP2*, *RASSF8*, *RBBP4*, *ONECUT2*, *PTEN*	2.47	0.0117	3.19	0.00006
hsa-miR-455-3p	*UBA7*, *NCSTN*, *RAB18*	5.51	0.00515	4.09	0.03758

FR—Fold regulation; target genes were searched at miRTargetLink 2.0 (https://ccb-compute.cs.uni-saarland.de/mirtargetlink2/ accessed on 1 November 2024) and filtered with criteria “strongly validated”.

## Data Availability

The datasets generated during and/or analysed during the current study are available from the corresponding author upon reasonable request.
